# Topological Phase Transition in Two-Dimensional Magnetic Material CrI_3_ Bilayer Intercalated with Mo

**DOI:** 10.3390/ma18204751

**Published:** 2025-10-16

**Authors:** Chen-En Yin, Angus Huang, Horng-Tay Jeng

**Affiliations:** 1 Department of Physics, National Tsing Hua University, Hsinchu 30013, Taiwan; shawnyin60@gmail.com (C.-E.Y.);; 2 Physics Division, National Center for Theoretical Sciences, Taipei 10617, Taiwan; 3 Institute of Physics, Academia Sinica, Taipei 11529, Taiwan; 4 Research Center for Semiconductor Materials and Advanced Optics, Chung Yuan Christian University, Taoyuan 32031, Taiwan

**Keywords:** 2D topological material, first-principle calculation, magnetism, quantum anomalous Hall effect, topological phase transitions

## Abstract

Motivated by the seminal discoveries in graphene, the exploration of novel physical phenomena in alternative two-dimensional (2D) materials has attracted tremendous attention. In this work, through theoretical investigation using first-principles calculations, we reveal that Mo-intercalated ${\mathrm{CrI}}_{3}$ bilayer exhibits ferromagnetic semiconductor behavior with a small easy-plane magnetocrystalline anisotropy energy (MAE) of 0.618 meV/Cr(Mo) between (100) and (001) magnetizations. The spin–orbit coupling (SOC) opens a narrow band gap at the Fermi level for both magnetization orientations with nonzero Chern number for realizing the quantum anomalous Hall effect (QAHE) in the former and with trivial topology in the latter. The small MAE implies the efficient experimental manipulation of magnetization between distinct topologies through an external magnetic field. Our findings provide compelling evidence that the QAHE in this system originates from the quantum spin Hall effect (QSHE), driven by intrinsic magnetism under broken time-reversal symmetry. These unique properties position Mo-intercalated ${\mathrm{CrI}}_{3}$ as a promising candidate for tunable spintronic applications.

## 1. Introduction

In recent years, two-dimensional (2D) magnetic materials have attracted marvellous research interest, leading to a large number of experimental and theoretical studies [1,2]. These materials, ranging from graphene with twisted angles between its layers to transition metal dichalcogenides with enhanced properties, hold promise as fundamental components in future technological advancementsi [3,4,5]. An advantage of these materials is their dissipationless edge states, which are expected to significantly enhance transport performance [6,7,8,9]. By combining tunable exchange interactions with topological band structures, 2D magnetic topological materials are poised to become foundational building blocks for next-generation, low-power spintronic devices [10,11,12]. Van der Waals heterostructures, in particular, provide a versatile platform for engineering and exploiting emergent quantum phases [13].

Spin-gapless semiconductors (SGS) represent a unique category of materials that bridge the domains of zero-gap materials and half-metals [14]. Their band structures feature two bands crossing one another at the Fermi level, which can be characterized as either Dirac-type or parabolic-type, thus rendering them gapless. In many cases, these materials exhibit the emergence of a band gap under spin-orbit coupling (SOC), leading to semiconductors with topological characteristics. Typically, within the framework of magnetization order, SGS materials are often ferromagnetic, exhibiting high Curie temperatures [15,16,17,18,19,20]. Furthermore, Dirac-type SGS materials are frequently associated with relatively high electron mobility [17,21,22].

The topological effect constitutes a crucial property in 2D materials. The Hall effect, first discovered by Edwin Hall in 1879, describes the behavior of current flow in the presence of an external magnetic field, contingent upon the material’s electrical properties [23]. The anomalous Hall effect (AHE) arises from the Hall effect when current flow is induced spontaneously without the application of an external magnetic field [24,25]. When the anomalous Hall conductance reaches quantization, it leads to the quantum anomalous Hall effect (QAHE) [26,27]. The QAHE is topologically protected and is typically characterized by a non-zero Chern number and the presence of chiral edge states within a global bulk band gap [28]. Induced by intrinsic magnetic moments and generally associated with spin-orbit coupling, the QAHE has predominantly been observed in ferromagnetic materials exhibiting time-reversal symmetry breaking [28,29,30]. The chiral edge states, which serve as carriers of quantized current, are intrinsically linked to the ferromagnetic property of these materials [31].

The quantum spin Hall effect (QSHE) represents another category within the Hall effect paradigm; however, it manifests differently from the QAHE [32]. In the QSHE, there exist two counter-propagating chiral currents that depend on spin direction, contrasting with the single chiral current flow observed in the QAHE. The QSHE is characterized by two distinct surface or edge states that intersect within a band gap, resulting in a net Chern number of zero due to the opposing contributions of these states [33,34,35,36,37]. The presence of ferromagnetic properties in the material can disrupt the QSHE [38]. Specifically, the breaking of time-reversal symmetry results in the retention of only one state with compatible chirality, while the opposing state becomes fragmented [39]. This phenomenon presents a potential pathway for the generation of QAHE from QSHE.

The quantum anomalous Hall effect (QAHE) has primarily been observed in bulk materials such as ${\mathrm{HgCr}}_{2}{\mathrm{Se}}_{4}$ [40], $Y_{2}{\mathrm{Ir}}_{2}O_{7}$ [41], and various Heusler alloys [42,43]. Recently, several 2D materials exhibiting QAHE have been identified. Notable examples include the chiral edge states observed in the MnNF/MnNCl heterojunctions with ferromagnetic order [44], monolayer transition metal dichalcogenides under all-optical design [45], photonic crystals [46], and Weyl semimetal ${\mathrm{Co}}_{3}{\mathrm{Sn}}_{2}S_{2}$ [47], all of which have attracted considerable interest.

Recent studies have demonstrated that external modulation strategies, such as Floquet engineering and heterostructure design, provide effective routes to induce nontrivial topological phases in two-dimensional materials [48,49,50,51,52,53,54,55,56,57]. These approaches enable functionalities ranging from topological phase transitions and superlubricity to negative differential resistance, integrated sensing-memory-computing, and topological photonic states. Furthermore, device performance can be enhanced by suppressing magnetic noise through multilayer or laminated composite shielding techniques [58,59].

${\mathrm{CrI}}_{3}$ has been synthesized and experimentally characterized, revealing that it is a van der Waals layered material with a variety of magnetic phases [60,61,62]. While ${\mathrm{CrI}}_{3}$ displays ferromagnetic order in its bulk state, it exhibits interlayer antiferromagnetic behavior in thin films [63,64,65]. However, a significant limitation of ${\mathrm{CrI}}_{3}$ is the substantial gap between its Fermi level, which poses challenges for practical applications [66]. Other research has intercalated atoms into the interlayer space to modify the physical properties in ${\mathrm{CrI}}_{3}$, such as self-intercalation to enhance the magnetic moment [67], intercalated oxygen to flip the magnetization [68], intercalated copper to achieve coexistence of ferroelectricity and ferromagnetism [69], intercalated copper and silver to induce polarization with interlayer ferromagnetism [70], and intercalated transition metals to control the magnetic order [71]. Similar manipulations can also be found in transition metal dichalcogenides [72,73,74,75].

## 2. Materials and Method

**Figure 1:**
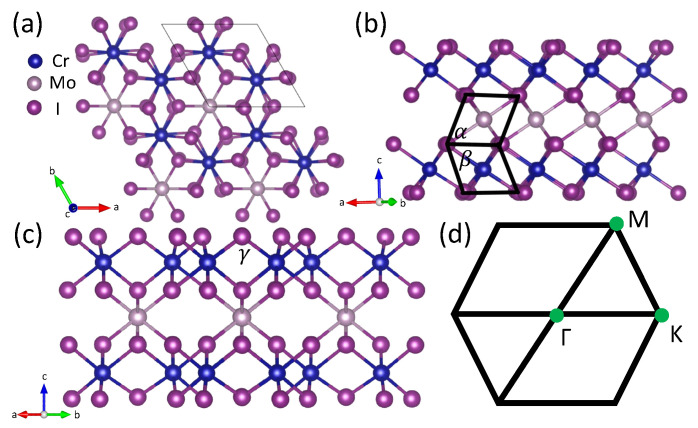
(**a**) Top view of bilayer ${\mathrm{CrI}}_{3}$ intercalated with Mo atoms. (**b**,**c**) Side views of bilayers ${\mathrm{CrI}}_{3}$ intercalated with Mo atoms and the bond angle for different directions. (**d**) The first Brillouin zone with high symmetry points.

Intercalation is a common technique used in both theoretical and experimental research; this manipulation has the potential to reveal unconventional properties like topology [76,77,78,79,80,81,82,83,84,85,86]. In this study, we present the discovery of a new 2D QAHE material with ferromagnetic order: ${\mathrm{CrI}}_{3}$ bilayer intercalated with Mo atom as shown in Figure 1. When the magnetization is oriented in the out-of-plane direction (001), this material demonstrates a clean topological edge state traversing the global band gap, alongside spin-gapless semiconductor (SGS) characteristics that are associated with high Curie temperatures and, in some instances, elevated mobilities. Notably, when the magnetization is manipulated to an in-plane direction (100), the system transitions to a non-topological state within the SGS framework, demonstrating that the topological phase can be controlled by alternating the magnetization orientation.

Molybdenum was selected as the intercalant because Mo-based transition metal dichalcogenides (TMDs) exhibit tunable electronic properties [87,88,89,90,91,92,93,94,95], and ${\mathrm{MoI}}_{3}$ has already been demonstrated to be both theoretically and experimentally stable [96,97]. We suggest that intercalation represents another essential approach for achieving topological phase transitions. Prior studies have also verified the feasibility of intercalating transition metals into TMD materials [73], suggesting that experimental realization of Mo intercalation in ${\mathrm{CrI}}_{3}$ is plausible despite being in the theoretical stage at present [67,68,69,70,71].

First-principles electronic structure calculations were performed using the Vienna Ab initio Simulation Package (VASP) [98,99,100,101] based on density functional theory (DFT) [102] with projected augmented wave (PAW) potential [103]. A vacuum thickness of 15 Å with an energy cutoff of 400 eV was adopted for the ${\mathrm{CrI}}_{3}$ bilayer intercalated with Mo system. A 6 × 6 × 1 Γ-point centered Monkhorst-Pack [104] k-point over the Brillouin zone was used for geometrical relaxation, while a 12 × 12 × 1 k-point mesh was used for total energy calculations. An on-site Hubbard *U*-value of 3.0 eV for both Cr and Mo atoms was used in the generalized gradient approximation plus Hubbard U method (GGA+U) [105,106]. The structures were fully relaxed until the total energy and atomic force converged to $10^{-6}$ eV and 0.01 eV/Å, respectively. The Wannier function for the Cr 3d, Mo 4d, and I 5p orbitals were usded in Wannier90 code [107,108,109,110] with initial projections set to the spherical harmonics $Y_{2m}\left(m=-2,-1,0,1,2\right)$ and the WannierTools package [111] was used to calculate the Wannier charge center and edge states properties.

By intercalating Mo atoms between bilayer ${\mathrm{CrI}}_{3}$ in this work, a material with enhanced properties can be achieved compared to pure ${\mathrm{CrI}}_{3}$. The resulting bilayer exhibits a hexagonal lattice structure, with Mo atoms intercalated within the interstitial spaces of ${\mathrm{CrI}}_{3}$, as illustrated in Figure 1a. This configuration resembles an insertion into the honeycomb lattice from a top–down perspective while forming ${\mathrm{MoI}}_{3}$ within the van der Waals gap between two ${\mathrm{CrI}}_{3}$ layers, as shown in Figure 1b. The intercalation of Mo atoms induces structural distortions, reducing the lattice symmetry to the triclinic space group P1. Iodine atoms in the inner layer are drawn closer to the Mo atoms, while those in the outer layer are pushed away, resulting in distortion as depicted in Figure 1c. The angle γ, which is nearly perpendicular in the original ${\mathrm{CrI}}_{3}$ structure, is measured as $97.4^{\circ }$ due to the influence of Mo, as detailed in Table 1. The angle between adjacent iodine atoms (Figure 1) indicate that two distinct quadrilateral formations are oriented in different directions.

## 3. Results and Discussion

**Figure 2:**
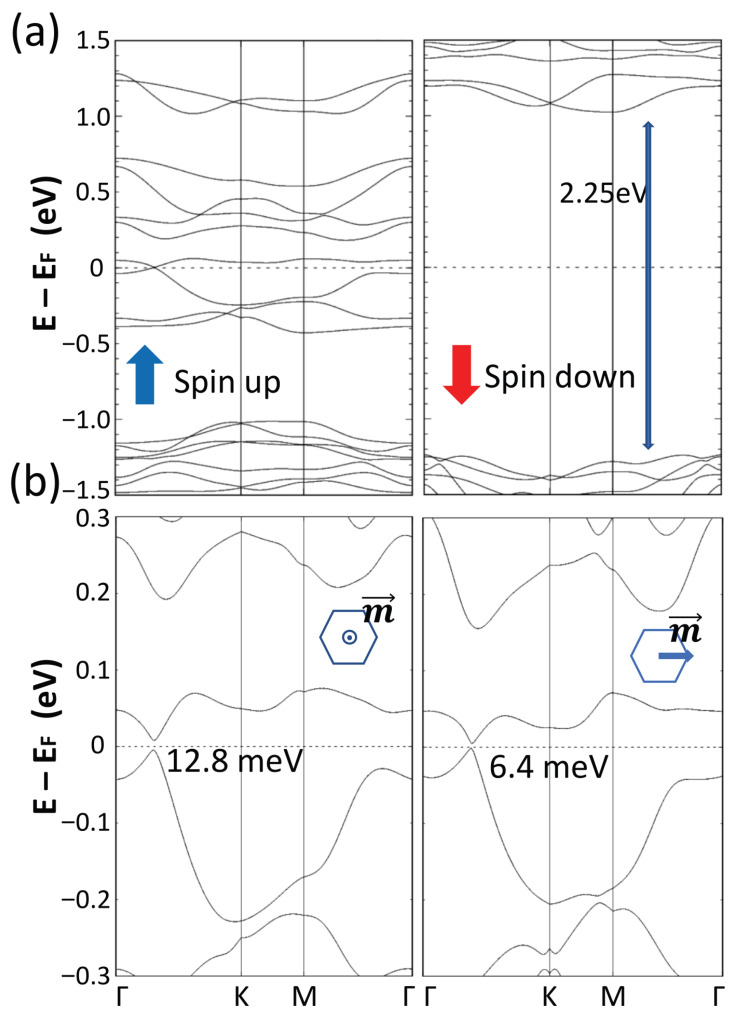
(**a**) Spin-resolved band structure of bilayer ${\mathrm{CrI}}_{3}$ intercalated with Mo atoms along high symmetry k-points without SOC in ferromagnetic order. (**b**) Band structure of bilayer ${\mathrm{CrI}}_{3}$ intercalated with Mo. SOC is included self-consistently with the magnetization ($\vec{m}$) pointing toward (001) direction and (100) direction, as indicated in the insets.

Various magnetic orders have been explored to ascertain the ground state of this material. All forms of antiferromagnetic order studied in this work result in significantly higher energy states and hence are not discussed. Figure 2a shows the spin-resolved band structure of the ferromagnetic ground state. A Dirac cone with the Dirac point at the Fermi level along ΓK path in the spin up channel can be seen clearly, whereas in the spin down channel, an energy gap of 2.25 eV exists at the Fermi level, resulting in a rarely found half-metallic Dirac electronic band structure. Magnetization in the (100) direction corresponds to the ground state exhibiting ferromagnetic order, whereas magnetization in the (001) direction possesses slightly higher energy of 0.618 meV/Cr(Mo). With spin-orbit coupling (SOC) taken into consideration, a Dirac-like cone featuring a narrow gap at the Dirac point along the Γ−K path of 12.8 meV and 6.4 meV is observed for the (001) and (100) magnetization, respectively, as illustrated in the accompanying Figure 2b. An additional cone is evident along the trajectory towards the ˘K point from Γ due to geometric symmetry, while the magnitudes of the two gaps arising from the different magnetic orders are smaller than the thermal energy at room temperature.

**Figure 3:**
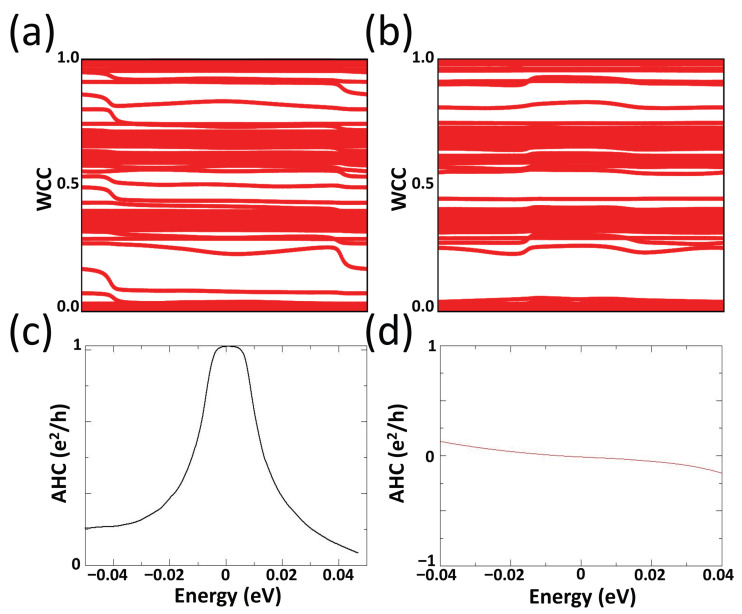
(**a**,**b**) Wannier charge center (Wilson loop) calculation from the Wannier function in the momentum space with magnetization along (001) and (100) direction, respectively. (**c**,**d**) Anomalous Hall conductivity calculation around the Fermi level for magnetization along the (001) and (100) direction, respectively.

Subsequent analysis employing Wannier calculations was conducted to investigate the physical properties within the gap as illustrated in Figure 3. Utilizing the Wannier charge center (WCC), sometimes referred to Wilson loop, calculations for magnetization along (001) direction, we identified a line traversing from zero to one without self-intersection on the trajectory depicted in Figure 3a. The path extending from the bottom right to the top left signifies the presence of a positive Chern number for each trajectory [112]. The calculation of the anomalous Hall conductivity (AHC), illustrated in Figure 3c, confirms that this material exhibits QAHE when the magnetization is perpendicular to the surface plane, achieving an anomalous Hall conductivity of one unit $\frac{e^{2}}{h}$ at the Fermi level, resulting in a Chern number of one for the (001) magnetization order. Conversely, as evidenced in Figure 3b for magnetization along (100) direction, there is no trajectory passing from zero to one, indicative of a zero Chern number state. This corresponds to the disappearance of anomalous Hall conductivity at the Fermi level, as portrayed in Figure 3d when the magnetization is aligned within the (100) surface plane.

**Figure 4:**
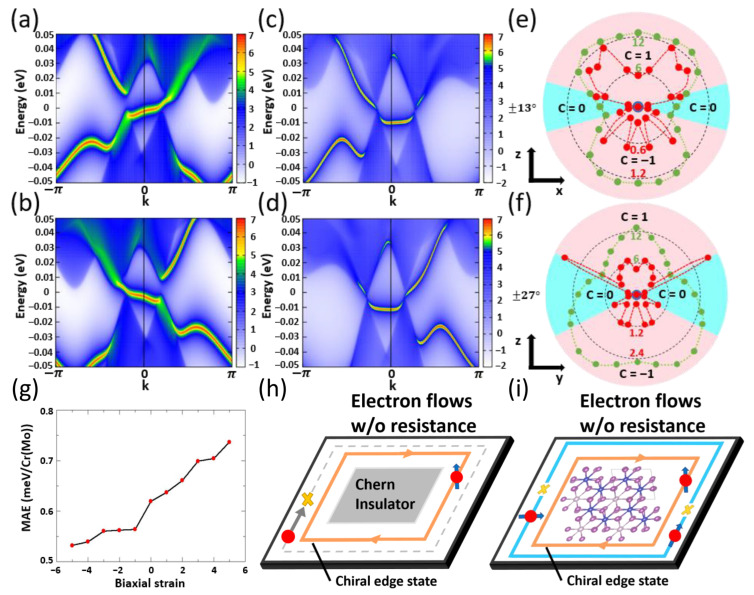
Edge-state calculations using the Wannier function for the ribbon composed of ${\mathrm{CrI}}_{3}$ bilayer intercalated with Mo. (**a**) Magnetization along the (001) direction, (**b**) magnetization along the (00-1) direction, (**c**) magnetization along the (100) direction, (**d**) magnetization along the (−100) direction. Phase diagram of the Chern number as a function of polar angle θ of the magnetization on the *x*-*z* plane (**e**) and on the *y*-*z* plane (**f**). The green polar radius indicates the value of band gap in meV (green mumbers). The red polar radius indicates the total energy in meV/Cr(Mo) (red numbers). (**g**) Magnetic anisotropy energy under the biaxial strain. (**h**) Schematic sketch of the chiral edge state of a Chern insulator. The orange (gray) line shows the existence (absence) of the chiral edge state in a Chern insulator. (**i**) Schematic sketch of the tunable chiral edge state on a chip composed of ${\mathrm{CrI}}_{3}$ bilayer intercalated with Mo in this work. The orange and blue lines show respectively the existence and absence of the chiral edge state tunable through flipping the out-of-plane (001) to the in-plane (100) magnetization by applying external magnetic field.

With (001) magnetization, Figure 4a reveals the existence of a single chiral edge state that traverses the energy gap from the Dirac cone in the −KG path to the Dirac cone in the GK path, specifically from the lower left to the upper right. This edge state, identified as the chiral edge state, is a clear manifestation of a nontrivial Chern number of 1. Furthermore, a second chiral edge state extending from the upper left to the lower right through the energy gap is shown in Figure 4b for the (00-1) magnetization evidences the negative topological Chern number of −1.

The intersection of these two edge states within the gap would contribute to the QSHE [34]. However, in this context, one of the edge states is disrupted due to the breaking time-reversal symmetry, which arises from the intrinsic magnetization of the material. This intrinsic magnetization damages one of the chiral edge states with incompatible chirality. Upon rotation of the magnetization from (001) to the opposite (00-1) direction, as illustrated in Figure 4b, the previously fractured in-gap topological edge state reconnects with the other one destroyed. This behavior is a direct consequence of the established intrinsic magnetization. Therefore, this QAHE associated with the chiral edge state can be understood as stemming from the time-reversal symmetry breaking inherent to the QSHE. On the other hand, with magnetization along (100) and (−100) direction, Figure 4c,d show a trivial edge state that interlinks the two Dirac cones in the conduction band, revealing topologically trivial zero Chern number phases for in-plane magnetization orientations.

Previous studies reported that the topological features in monolayer ${\mathrm{CrI}}_{3}$ are located far from the Fermi level, with a global band gap of approximately 1 eV [113,114]. Consequently, electron or hole doping is typically required to access its topological properties [114]. In multilayer ${\mathrm{CrI}}_{3}$, interlayer antiferromagnetic ordering further suppresses the conditions necessary for QAHE. By contrast, the intercalation approach proposed here enables the realization of topological phases without the need of doping.

To explore the minimum angle required for tuning the topological trivial zero Chern number to nontrivial Chern number of ±1 phase by rotating the magnetization direction from (100) to (100), Figure 4e shows the phase diagram of Chern number with magnetization on the x−z plane. The phase diagram demonstrates a critical angle of $\pm 13^{\circ }$ for the magnetization to achieve adequate intrinsic magnetization in the out-of-plane direction, thereby contributing to the emergence of the QAHE. In comparison, the dependence of the polar angle of the magnetization in the y−z plane presented in Figure 4f shows, to some extent, different behavior with a larger critical angle of $\pm 27^{\circ }$. The distinct phase transition angle observed across the two orthogonal planes exemplifies the topological warping effect [115]. The phenomenon, which enables the controllable tuning of Chern numbers through variations in magnetization direction, has been documented in monolayer transition metal oxides and chiral magnets [116,117]. In the former, the Chern number can transit between low and high phases, whereas chiral magnets exhibit tuning capabilities between ±2 and the trivial phase. Notably, in this study, the Chern number can be manipulated between ±1 and a relatively extensive range of trivial phases, rendering it advantageous for applications in spintronics. The stability observed in transitioning through critical points across a broad spectrum of trivial phases distinguishes two opposite non-trivial phases.

The trend in band gap values versus magnetization polar angle (green curves in Figure 4e,f) observed here deviates from findings in earlier research, as a persistent band gap exists even in the trivial phases where the band gap is reported to be absent [116,117]. The band gap reaches the minimum value around zero polar angles but is not confined to zero polar angle, attributed to disorder stemming from material distortion; nevertheless, the overarching trend remains comparable to that documented in extant literature [118]. Tuning the Chern number with the orientation of the magnetization has also been observed in experiments [119,120]. On the other hand, the total energy as a function of the magnetization polar angle (red curves in Figure 4e,f) shows the easy plane magnetic anisotropy and the energy required to rotate the (100) magnetization to (001) magnetization, i.e., the energy required for tuning the topological phases among zero and ±1 Chern numbers. The local energy minima around the horizontal and vertical polar angles achieve the thermal stability of both the topologically trivial and nontrivial phases, respectively, leading to high advantages for real applications in nano-scale devices.

Moreover, the effect of biaxial strain, ranging from −5% to 5%, on magnetic anisotropy energy as depicted in Figure 4g illustrates a decline in magnetic anisotropy energy as the biaxial strain transitions from tensile to compressive. Tuning in MAE is essential because a high MAE may cause tuning difficulties, while a low MAE might lead to unavoidable heat fluctuations. The strain-dependent MAE presented here serves as a guide for designing a tunable topological edge state in a nano-device, which can be approach by the electric field [121]. The chiral edge state in a Chern insulator as depicted in Figure 4h illustrates that electrons exhibiting compatible spin chirality with an external magnetic field will generate an edge state current when this field is applied. In certain circumstances, intrinsic magnetization can be regarded as functionally analogous to the characteristics of the external magnetic field [122]. Conversely, in the absence of either an external magnetic field or intrinsic magnetization, no edge current will be present. However, as illustrated in Figure 4i, the material under investigation demonstrates a chiral edge state that facilitates edge current on a chip composed of ${\mathrm{CrI}}_{3}$ bilayer intercalated with Mo when the intrinsic magnetization is oriented along the (001) direction. In contrast, the chiral edge state becomes disrupted when the magnetization is oriented in the in-plane direction. The existence and absence of the chiral edge state is tunable through flipping the out-of-plane (001) to the in-plane (100) magnetization by applying an external magnetic field or the electric-field-induced magnetization control [123]. Such nuanced characteristic present advantages for applications in tunable spintronics.

## 4. Conclusions

In conclusion, this study demonstrates a nontrivial topological effect in the ${\mathrm{CrI}}_{3}$ bilayer intercalated with Mo through ab-initial calculations. This material exhibits the capacity of topological phase transition between two ferromagnetic phases with (001) and (100) magnetization via the application of an external magnetic field while maintaining small magnetocrystalline anisotropy energy. Specifically, the (001) magnetization configuration exhibits a nonzero Chern number, characteristic of a topological spin-gapless semiconductor with chiral edge states—a prerequisite for realizing the quantum anomalous Hall effect. In contrast, the (100) configuration is topologically trivial. The small MAE suggests that external magnetic fields can efficiently manipulate the magnetization, enabling transitions between topologically trivial (zero Chern number) and nontrivial (nonzero Chern number) phases. The capability to manipulate the topological phase with a Chern number of ±1 and 0 by varying magnetization polar angles holds significant promise for dissipation-less spin transport applications.

## Figures and Tables

**Table 1:** Lattice constant, interlayer distance, bond angle between adjacent iodine atoms (Figure 1), magnetocrystalline anisotropy energy between magnetization in the (001) direction and the (100) direction with ferromagnetic order in millielectron volt per magnetic atom (meV/Cr(Mo)) of bilayer ${\mathrm{CrI}}_{3}$ intercalated with Mo.

*a*(*Å*)	*d*(*Å*)	α(θ)	β(θ)	γ(θ)	MAE(meV/Cr(Mo))
7.24	6.67	59.2	55.9	97.4	0.618

## Data Availability

The original contributions presented in this study are included in the article. Further inquiries can be directed to the corresponding author.
